# Bioactive Phytoconstituents as Potent Inhibitors of Tyrosine-Protein Kinase Yes (YES1): Implications in Anticancer Therapeutics

**DOI:** 10.3390/molecules27103060

**Published:** 2022-05-10

**Authors:** Chunmin Yang, Afsar Alam, Fahad A. Alhumaydhi, Mohd Shahnawaz Khan, Suliman A. Alsagaby, Waleed Al Abdulmonem, Md. Imtaiyaz Hassan, Anas Shamsi, Bilqees Bano, Dharmendra Kumar Yadav

**Affiliations:** 1 School of Engineering, Guangzhou College of Technology and Business, Guangzhou 510850, China; hbycmar@163.com; 2 Department of Computer Science, Jamia Millia Islamia, Jamia Nagar, New Delhi 110025, India; afsar2004011@st.jmi.ac.in; 3 Department of Medical Laboratories, College of Applied Medical Sciences, Qassim University, Buraydah 52571, Saudi Arabia; f.alhumaydhi@qu.edu.sa; 4 Department of Biochemistry, College of Sciences, King Saud University, Riyadh 11451, Saudi Arabia; moskhan@ksu.edu.sa; 5 Department of Medical Laboratory Sciences, College of Applied Medical Sciences, Majmaah University, Majmaah 11932, Saudi Arabia; s.alsaqaby@mu.edu.sa; 6 Department of Pathology, College of Medicine, Qassim University, P.O. Box 6655, Buraydah 52571, Saudi Arabia; waleedmonem@qumed.edu.sa; 7 Centre for Interdisciplinary Research in Basic Sciences, Jamia Millia Islamia, Jamia Nagar, New Delhi 110025, India; mihassan@jmi.ac.in (M.I.H.); anas.shamsi18@gmail.com (A.S.); 8 Centre of Medical and Bio-Allied Health Sciences Research, Ajman University, Ajman P.O. Box 346, United Arab Emirates; 9 Department of Biochemistry, f/O Life Science, Aligarh Muslim University, Aligarh 202002, India; 10 College of Pharmacy, Gachon University of Medicine and Science, Hambakmoeiro, Yeonsu-gu, Incheon 21924, Korea

**Keywords:** YES1 kinase, cancer, phytoconstituents, drug discovery, Glabrene, Lupinisoflavone C, molecular dynamics simulations, free energy landscape

## Abstract

Tyrosine-protein kinase Yes (YES1) belongs to the Tyrosine-protein kinase family and is involved in several biological activities, including cell survival, cell–cell adhesion, cell differentiation, and cytoskeleton remodeling. It is highly expressed in esophageal, lung, and bladder cancers, and thus considered as an attractive drug target for cancer therapy. In this study, we performed a virtual screening of phytoconstituents from the IMPPAT database to identify potential inhibitors of YES1. Initially, the molecules were retrieved on their physicochemical properties following the Lipinski rule of five. Then binding affinities calculation, PAINS filter, ADMET, and PASS analyses followed by an interaction analysis to select safe and clinically better hits. Finally, two compounds, Glabrene and Lupinisoflavone C (LIC), with appreciable affinities and a specific interaction towards the AlphaFold predicted structure of YES1, were identified. Their time-evolution analyses were carried out using an all-atom molecular dynamics (MD) simulation, principal component analysis, and free energy landscapes. Altogether, we propose that Glabrene and LIC can be further explored in clinical settings to develop anticancer therapeutics targeting YES1 kinase.

## 1. Introduction

Tyrosine-protein kinase Yes (YES1) belongs to the Tyrosine-protein kinase family and subfamily SRC and functions in the AKT-mediated regulator signaling pathway and cell migration [1]. It is involved in several biological activities such as cell survival and cell growth, cell–cell adhesion, cell differentiation, cytoskeleton remodeling, etc. [2]. YES1 expresses at the broad spectrum in tissues such as the epithelial cells of the stomach and the proximal renal tubules, bone marrow, and spleen in developing fetus [3], expresses differently in adult epithelial cells of the proximal renal tubules and is keratinized in the basal epidermal layer of the epidermis. YES1 is highly expressed in different mammalian neurons, platelets, epithelial, and spermatozoan cells [4]. Studies have shown that *YES1* gene amplification exists in many cancers such as oesophagal [5], lung [6], bladder cancer, etc.; this indicates kinase YES1 is an appealing target in anticancer therapy. 

The YES1 protein is a long polypeptide made up of 543 amino acid residues that contain an N-terminal and a disordered region (residues 1–45), a SH3 domain (residues 91–152), an SH2 domain (residues 158–255), and a C-terminal kinase domain (residues 277–530) [7]. The nucleotide-binding region comprises residues 283–291, whereas Lys305 is an ATP- binding site and Asp396 is the active site of YES1 [8]. The YES1 protein has a distinctive binding pocket which makes it a suitable target in anticancer therapy. Moreover, its structure available from the AlphaFold can provide a valuable insight into the design of selective and highly potent ATP-competitive inhibitors (https://alphafold.ebi.ac.uk/entry/P07947; accessed on 3 January 2022).

Nowadays, virtual screening-based studies have become crucial for computer-aided drug design and discovery [9]. Molecular docking-assisted virtual screening makes it easy to find ligands that could bind the target receptor functionally and precisely. This technique is one of the most successful techniques to identify high-affinity binding ligands to target receptor proteins. It is a computer-based screening method to screen different chemical compounds available from many chemical databases for the identification of potential drug-like compounds. In the computer-aided drug design process, virtual screening by the molecular docking process is also combined with various other filters such as the Lipinski RO5 violation rule [10], PAINS filter [11], ADMET properties, and PASS analysis [12]. These methods play a crucial role in drug discovery processes. 

In this study, we have taken a library of 5875 phytochemical compounds from the IMMPAT database after applying the Lipinski RO5 violation value ‘zero’ [13]. The IMMPAT is the largest free curated database on the phytochemicals of Indian medicinal plants for virtual screening. We obtained the three-dimensional structure of YES1 from the Alphafold database [14]. After that, we performed a virtual screening of these compounds against YES1 to find its high-affinity binding partners using InstaDock [15]. Based on the predicted binding modes and score values, we selected the top hits, and then we ran SwissADME to filter the compounds with no PAINS patterns. After the PAINS filter, ADMET properties were calculated through the pkCSM server [16]. Finally, based on the specific interactions, we selected those compounds which bound specifically towards the binding site of YES1.

## 2. Results and Discussion

### 2.1. Molecular Docking-Based Virtual Screening

Molecular docking of phytochemical compounds from the IMPPAT database was conducted to find their binding affinities with YES1. The InstaDock generated binding affinities and docked conformations for each compound in the library which had a total of 5875 compounds after applying the RO5 filter. After the molecular docking process, another filter was applied to the compounds based on their binding affinity with YES1. The selected compounds had a significant docking score with the binding site of YES1. We selected the top 30 hits from 5875 phytochemicals with a binding affinity ≤ −9.7 kcal/mol with YES1 (Table 1). Here, most of the elucidated compounds were shown to have a higher affinity than the Dasatinib, a reference inhibitor of YES1. The results suggested that the selected phytochemicals compounds had a significant binding affinity with YES1, which could be further investigated to check the compound’s therapeutic potential in the drug discovery and development process.

### 2.2. ADMET Properties

ADMET properties are depicted based on a set of rules based on the pharmacokinetic properties of chemical compounds. The phytochemical compounds selected after docking were subjected to screening for ADMET properties. The best two compounds out of 30 hits were selected after ADMET prediction, which had good ADMET properties without any toxic patterns (Table 2). The elucidated compounds had similar ADMET properties; consequently, both were selected for further analysis.

### 2.3. PASS Analysis

To ensure the effectiveness of compounds with the desired properties, the biological properties of an elucidated compound must be investigated. PASS analysis was performed in this study to investigate the biological activity of the elucidated compounds. The biological properties of both compounds are mentioned in Table 3, with their scores as active or inactive. The results suggest that the compounds Glaberene and Lupinisoflavone C (LIC) had antineoplastic, TP53 expression enhancer, and chemopreventive features with convincing Pa values, i.e., between 0.716 and 0.896. This suggests that the elucidated compounds, i.e., Glaberene and LIC, possess great potential for use in anticancer therapies.

### 2.4. Interaction Analysis

**Figure 1:**
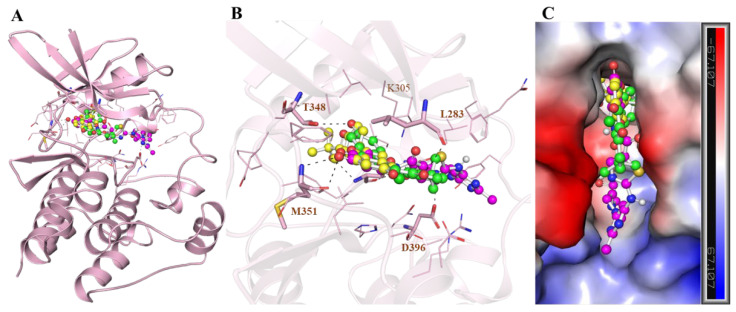
Molecular interactions of (**A**) YES1 with Glabrene (green), LIC (yellow), and Dasatinib (magenta) (**B**) Cartoon illustration of protein–ligand interactions. (**C**) Electrostatic potential surface view of YES1 bound with the selected compounds.

The interaction analysis was performed by splitting all the possible docking conformations from the out files of the docked Glabrene and LIC. The analysis found that Glabrene and LIC interact with common residues, including the ATP binding site of YES1, i.e., Lys305. The detailed binding pattern of Glabrene and LIC is illustrated in Figure 1. The figure shows that Glabrene and LIC interact closely with Lys305 (ATP binding site) of the YES1 protein, which is crucial for its activity (Figure 1B). The structural representation shows that Glabrene and LIC are bound into the deep binding pocket cavity of YES1 with a good complementarity (Figure 1C). 

**Figure 2:**
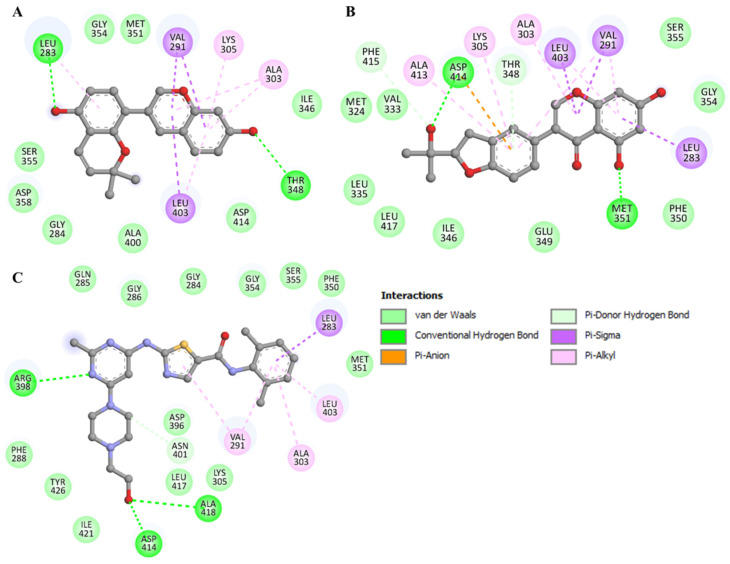
Representation of the 2D interaction showing YES1 interactions with (**A**) Glabrene, (**B**) LIC, and (**C**) Dasatinib.

The detailed interaction of both compounds was further explored for their interactions with the active/critical site residues of YES1. It is clear from Figure 2 that Glabrene and LIC share common interactions while interacting with the ATP-binding site residue of YES1. The ATP-binding site is essential for the functional activities of any kinase. The ATP-binding site is located at the main catalytic center of YES1, where Glabrene and LIC bind. Therefore, it indicates that Glabrene and LIC could act as the potential ATP competitive inhibitors of YES1. The chemical properties of the elucidated compounds are mentioned in Table 4.

### 2.5. MD Simulation

The MD simulation method is widely used to study the structural detail and dynamic behavior of protein–ligand complexes at the atomic level. Therefore, three systems, YES1-Glabrene, YES1-LIC, and the apo YES1 were studied in all-atom MD simulations for 100 ns. The stability and dynamics of YES1 in a complex with Glabrene and LIC were analyzed by exploring various systematic and structural parameters discussed below.

#### 2.5.1. Structural Dynamics and Compactness

**Figure 3:**
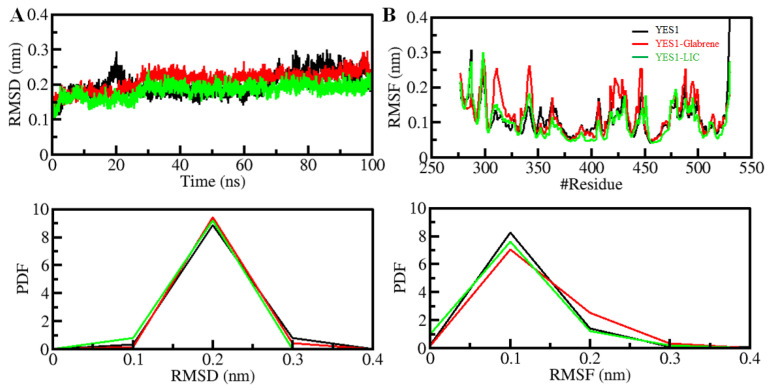
Structural dynamics of YES1 upon Glabrene and LIC binding. (**A**) RMSD plot of YES1 in complexed with Glabrene and LIC. (**B**) RMSF plot of YES1 and its complex with Glabrene and LIC. Lower panels show the probability distribution function of values as PDF. # represents number.

One of the most widely used parameters to study structural deviation in a protein is the root-mean-square deviation (RMSD). It is one of the most important methods to study the structural changes and dynamics of a protein structure [17,18]. The structural dynamics were assessed before and after the ligands bound to the protein. Here, we found average RMSD values of 0.20 nm, 0.21 nm, and 0.18 nm for YES1, YES1-Glabrene, and YES1-LIC, respectively (Table 5). The binding of Glabrene and LIC with YES1 indicated a good stability of the docked complexes which is equilibrated throughout simulation time as depicted from the RMSD graph (Figure 3A). For the YES1–Glabrene complex, a slight fluctuation was seen in the RMSD but without any shift. We observed that during the entire simulation period of 100ns, the RMSD of all systems was stabilized and balanced. The probability distribution function (PDF) for the distribution of RMSD values was also plotted, demonstrating the stabilization of YES1 with a high probability over compound binding (Figure 3A, lower panel).

To measure the residual vibrations in a protein structure during an MD simulation, the root means fluctuation (RMSF) has been proven to be a very helpful approach that tells us the impact of ligand binding on the residual fluctuations of the protein. By plotting the RMSF for each residue, we were able to study the residual dynamics of YES1 before and after ligand binding (Figure 3B). The protein–ligand system was remarkably reliable by reducing and stabilizing the RMSF fluctuation upon LIC binding. For Glabrene, the binding marginally increased in residual vibrations at some places, which indicated higher dynamics in the loop regions. In comparison, we could say that the YES1–LIC complex was more stable than the YES–Glabrene complex by comparing the RMSD and RMSF values. The PDF showed a decreased residual fluctuation in YES1 over the binding compounds (Figure 3B, lower panel).

**Figure 4:**
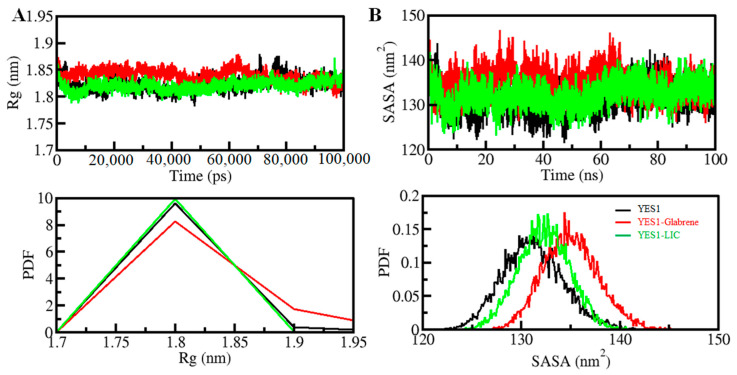
Structural compactness and folding of YES1 upon Glabrene and LIC binding. (**A**) *R_g_* plot and (**B**) SASA plot of YES1 with Glabrene and LIC. Lower panels show the probability distribution function values as PDF.

The radius of gyration (*R_g_*) is a useful parameter to study the compactness of a protein structure [17]. It is directly related to the folding and compactness of the protein structure. *R_g_* is the RMS distance from the set of atoms from their collective center of mass. To assess the compactness of the YES1–Glabrene and YES1–LIC complexes during the simulation time, *R_g_* was studied in time-evolution settings (Figure 4A). In the plot, the *R_g_* value slightly increased for the YES1–Glabrene complex, which can be related to the RMSD and RMSF values. The *R_g_* plot indicates that YES1 was folded stably in both complex forms. There was no change in the average *R_g_* value of YES1 upon LIC binding observed by the PDF analysis (Figure 4A, lower panel) (Table 5). 

The solvent-accessible surface area (SASA) of a protein is the surface area accessible to its adjacent solvent [19]. The stability and folding behavior of proteins are studied using SASA [20]. We found no changes in the SASA values at the entire simulation time by examining the plots, which indicates that the YES1–Glabrene and YES1–LIC complexes were quite stable (Figure 4B). Loose Packing of YES1 with Glabrene showed a slight increase in the SASA value, but it did not disturb the overall protein folding. Furthermore, the PDF analysis indicated a minor increase in the average SASA of YES1 with Glabrene complex during the simulation (Figure 4B, lower panel).

#### 2.5.2. Dynamics of Hydrogen Bonds

**Figure 5:**
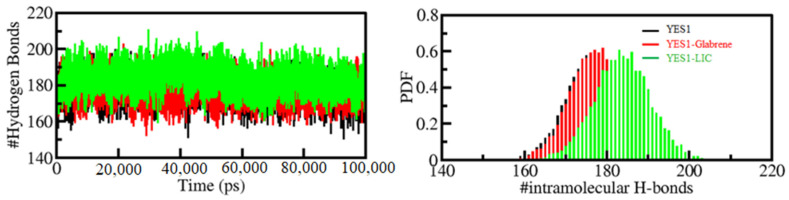
Hydrogen bond analysis. Time evolution of intramolecular H-bonds (**left panel**). The (**right panel)** shows the PDF of the hydrogen bond distribution. # represents number.

The hydrogen bond (H-bond) formation is crucial for protein folding dynamics. The breaking and making of H-bonds are key steps involved in the conformational dynamics of all proteins. To assess the reliability of intramolecular bonding in YES1–Glabrene and YES1–LIC complexes, the time evolution of H-bonds was analyzed. As the plot generated shows, there was no significant change in the number of H-bonds seen within YES1 when complexed with Glabrene and LIC. In YES1 intramolecularly, the average H-bonds formed before and after Glabrene and LIC complexes were found to be 177, 178, and 183, respectively (Figure 5, left panel). A slight increase in H-bonds was noticed, probably due to the increased compactness over the binding of the ligands. The PDF calculated for all three systems for intramolecular H-bonds showed good reliability (Figure 5, right panel). Concluded from the plots, the intramolecular H-bonds in YES1 showed stability throughout the simulation, even after the binding of the compounds.

**Figure 6:**
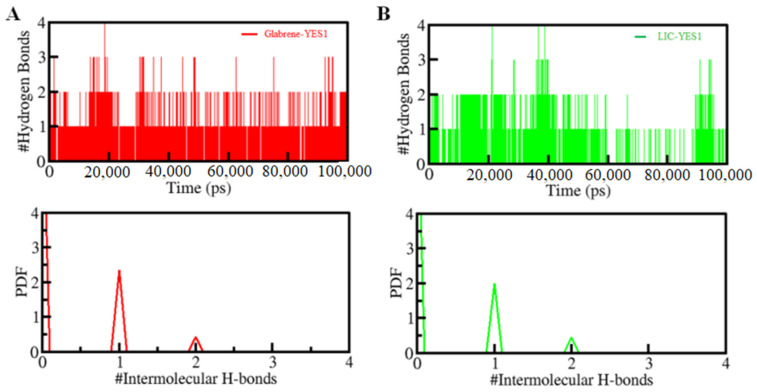
Time-evolution of intermolecular H-bonds formed within 0.35 nm between YES1 and (**A**) Glabrene and (**B**) LIC. The lower panels show the PDF of the hydrogen bond distribution. # represents number.

Moreover, the time evaluation of intramolecular H-bonds was further explored to determine the constancy of H-bonding in between the Glabrene and LIC with YES1. Within the Glabrene–YES1 and LIC–YES1, the average H-bonds formed were estimated to be one in each complex (Figure 6, upper panel). With the higher PDF value and the average number of H-bonding as one, the PDF suggested a fair constancy for the intramolecular H-bonds in both systems (Figure 6, lower panel). The Glabrene and LIC did not move from their initial docking position on YES1 as predicted from the intermolecular H-bonding, which stabilizes the complex structures.

### 2.6. PCA and FELs Analysis

**Figure 7:**
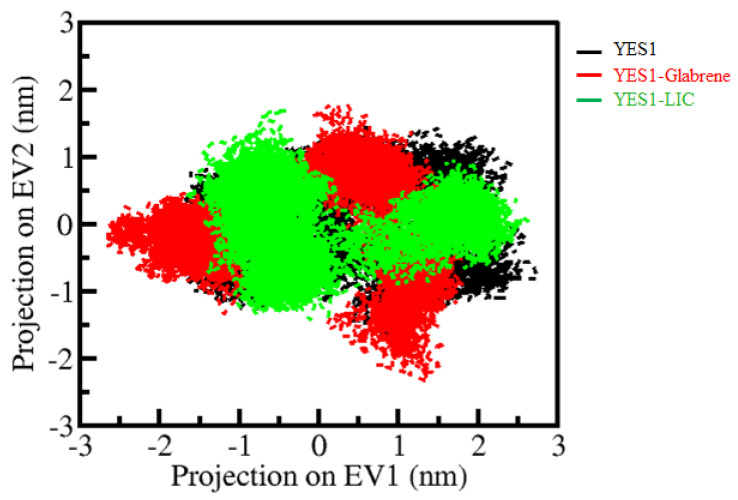
Principal component analysis. Two-dimensional conformational projections of YES1, YES1-Glabrene and YES1-LIC.

PCA is a useful approach for exploring the collective motions in protein and protein–ligand complexes. We used the essential dynamics approach to explore the conformational sampling of the YES1, YES1-Glabrene, and YES1-LIC complexes using simulated trajectories. The conformational sampling of YES1, YES1-Glabrene, and YES1-LIC in the essential subspace is shown in Figure 7. As projected, the conformations of YES1 on two different EVs were projected by its C_α_ atoms. The clusters of apo YES1 were covered by the YES1–Glabrene and YES1–LIC projections (Figure 7). The plot indicated that the YES1–Glabrene and YES1–LIC complexes occupied the same conformational space as YES1. It was depicted that the lesser flexibility conformational space covered by the YES1–LIC complex made it more stable than the YES1–Glabrene complex as shown inFigure 7.

**Figure 8:**
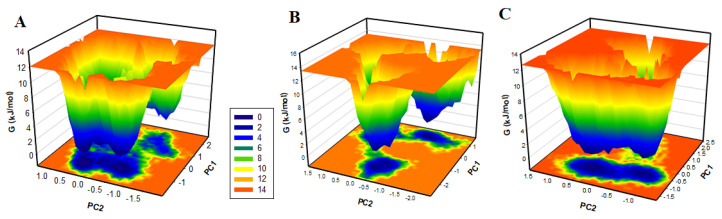
The free energy landscapes of (**A**) free YES1, (**B**) YES1-Glabrene, and (**C**) YES1-LIC.

The protein folding mechanism can be explored using FELs analysis. The FELs were generated to explore the global minima and conformational landscapes of the YES1, YES1–Glabrene and YES1–LIC systems. The FELs of YES1, YES1–Glabrene and YES1–LIC systems are illustrated in Figure 8. Deeper blue in FELs indicates the protein conformation with lower energy near to native states. YES1, when in the apo state has a single global minimum overall confined within a large basin (Figure 8A). The plot showed that the size and position of the phase confined within a single stable global minimum were slightly disturbed by the binding of YES1 with Glabrene and LIC, as suggested by the FEL plots of the complexes (Figure 8B,C). While binding with Glabrene and LIC, YES1 acquires different states with multiple basins but to overall global minima (Figure 8B,C). All-inclusive, the FELs suggested that the binding of Glabrene and LIC with YES1 did not lead to the unfolding of YES1 during the simulation.

## 3. Materials and Methods

### 3.1. Computer Environment and Web Resources

This study was carried out on the HPZ420 workstation running on Windows 10 OS. We used a high-speed wired ethernet internet connection and an uninterrupted power supply. Bioinformatics tools such as InstaDock [15] for docking-based virtual screening, PyMOL [21] for visualization and Discovery Studio Visualizer [22] for interactions and generating 2D plots were used. Various online web servers and databases such as UniProt [23], RCSB Protein Data Bank (PDB) [24], AlphaFold (Protein structure Database) [25], IMPPAT (Indian Medicinal Plants Phytochemistry and Therapeutics) [26], SwissADME [27], pkCSM [28], and PASS server [29] were used in this study for the extraction, assessment, and investigation of data. 

### 3.2. Receptor and Library Preparation

The three-dimensional structure of YES1 was taken from the AlphaFold database (https://alphafold.ebi.ac.uk/entry/P07947; accessed on 3 January 2022) and refined further using PyMOL by extracting the kinase domain from the whole coordinates. Energy minimization was performed to obtain a stable structure using the SWISS-PDB Viewer software (version 4.1.0; https://spdbv.unil.ch/; accessed on 4 January 2022) and validated through the UCLA webserver (SAVES v6.0). We used the Lipinski RO5 rule to filter and download the phytochemical compounds library from the IMPPAT database to ensure drug likeliness and biologically active compounds.

### 3.3. Molecular Docking Based Virtual Screening

Virtual screening and molecular docking have become very important in the drug discovery process as they reduce the time and cost of designing and delivering new therapeutics. It makes it easy to screen a large library of drug-like compounds against a predefined target, available at various free and commercial databases. In receptor-based virtual screening, we used the 3D structure of YES1 and phytoconstituents from the IMPPAT database to identify YES1 inhibitors. In this study, InstaDock was used for molecular docking-based virtual screening. We performed blind docking in which the entire receptor protein structure was used to find the best binding pocket by the compounds. The output files of InstaDock were in log-files and out-files, which were extracted based on the compounds’ binding affinity and docking conformations towards YES1 used for the further analyses.

### 3.4. ADMET Prediction

The extracted results after docking, we used ADMET properties to filter out the compounds. The ADMET properties and PAINS pattern (pan assay interference compounds) were assessed using swissADME and pkCSM webservers. The PAINS filter avoids compounds with a higher tendency to bind to multiple targets. ADMET properties help filter out compounds based on their absorption, distribution, metabolism, excretion, and toxicity, which is very important for a drug in passing clinical trials. Compounds with appreciable ADMET properties and no PAINS pattern were taken for further analysis.

### 3.5. PASS Analysis

PASS analysis is very valuable in a chemical–biological interactions study for assessing the biological properties of a chemical compound. We used the PASS server for the biological properties of the elucidated compounds after the ADMET filter. The PASS server gives results on two different labels, i.e., “probability to be active (Pa)” and “probability to be inactive (Pi)”. A higher Pa value indicates a higher probability for that associated property for the compound.

### 3.6. Interaction Analysis

The finally elucidated compounds were analyzed through PyMOL and Discovery Studio Visualizer for their detailed interactions with YES1. The out-files of the docked compounds were taken from the InstaDock output. Ribbon representation and electrostatic potential surface were generated through PyMOL. Hydrogen bonds formed within 3.5 Å were mapped in dotted lines and labeled. Two-dimensional plots of the interactions describing each type of interaction between the compounds and YES1 were generated through Discovery Studio Visualizer. 

### 3.7. MD Simulations

In a computer-aided drug discovery pipeline, MD simulation plays a crucial role in studying motions at the atomic level in protein–ligand structures [30]. The docking results of YES1 with the selected phytochemical compounds (Glabrene and LIC) were verified by MD simulation studies. We used GROMACS v5.5.1 to simulate the structural coordinates of YES1 and its docked complexes with Glabrene and LIC. It is widely used in the computer-aided drug design process and is an open-source software program for MD simulation. The PRODRG server [27] was used to create receptor–ligand complex topologies. For solvation, we placed each system in a cubic box at a distance of 10 Å from the center to the edges while utilizing the simple point charge (SPC216) water model. Moreover, the neutralization of the simulation system was performed by adding counterions (Na^+^ and Cl^−^) in an appropriate amount. Energy minimization was performed in the solvated system with the 1500 steps with the steepest descent approach followed by the conjugate gradient method to remove possible steric hindrances between the atoms. The two-step equilibration under the periodic boundary setting was carried out for 100 ps, at a constant volume with gradual heating from (0–300 K) temperature and the pressure of 1 atm. GROMACS inbuilt tools were used for the analysis described in our previous reports [31,32,33,34]. The Gromacs calculations were run on Lenovo ThinkSystem ST50 with Xeon^®^ processor.

### 3.8. Principal Component Analysis and Essential Dynamics

Principal component analysis (PCA) is a mathematical approach widely used to minimize data dimensionality [35]. This minimization is performed by categorizing directions, called principal components (PCs), along which the data variation is maximal. Generally, it uses only a few components to signify each sample through a few variables [36]. Besides, PCA can be used to uncover the differential motions of high amplitude in MD trajectories [35]. We analyzed the MD trajectories of YES1 before and after Glabrene and LIC binding for conformational sampling, atomic motions, and stability through PCA and free energy landscape (FEL) analysis [37].

## 4. Conclusions

Today, the world is persistently faced with several complex diseases such as cancer. The development of anticancer therapeutics based on modern settings is urgently needed. Targeting YES1 possesses anticancer efficacy for developing potential anticancer therapeutics. This work proposes the therapeutic management of cancer by using natural compounds targeting YES1. Here, we carried out an in silico analysis using state-of-the-art computational approaches. Two phytoconstituents, Glabrene and LIC, were identified as potential leads by assessing their physicochemical and drug-like properties and stable binding towards the AlphaFold predicted structure of YES1. The time-evolution results through the all-atom MD simulation and PCA and FEL analyses suggested a stable binding of elucidated compounds with YES1. Altogether, we recommend that Glabrene and LIC be further explored in in vitro and in vivo settings to develop anticancer therapeutics targeting YES1.

## Figures and Tables

**Table 1:** Binding affinity of the selected top 30 hits against YES1.

S. No.	Compound ID	Affinity (kcal/mol)
1	24,901,683	−10.9
2	5154	−10.6
3	102,267,534	−10.6
4	14,630,492	−10.6
5	146,680	−10.5
6	443,716	−10.5
7	101,651,627	−10.4
8	94,577	−10.3
9	442,851	−10.3
10	125,848	−10.2
11	11,438,278	−10.2
12	10,957,726	−10.2
13	9,798,203	−10.2
14	14,630,495	−10.2
15	5,245,667	−10.1
16	5,315,739	−10.1
17	5,281,809	−10.1
18	85,976,174	−10.1
19	53777-78-9	−10.0
20	97,679	−10.0
21	4737-28-4	−9.9
22	630,669	−9.9
23	633,072	−9.9
24	6,453,733	−9.9
25	11,035,494	−9.9
26	480,774	−9.8
27	104,860	−9.8
28	161,899	−9.8
29	10,144	−9.8
30	44,257,284	−9.7
31	Dasatinib	−9.7

**Table 2:** ADMET properties of the elucidated compounds.

Compound ID	Compound	Absorption	Distribution	Metabolism	Excretion	Toxicity
		*GI* *Absorption*	*BBB Permeation*	*CYP2D6* *Inhibitor*	*OCT2 Substrate*	*AMES*
480,774	Glabrene	High	0.068	No	No	No
44,257,284	LIC	High	−0.926	No	No	No

**Table 3:** Biological properties of the elucidated compounds predicted through the PASS server.

S.N	Compound	Pa	Pi	Activity
1.	Glabrene	0.896	0.006	HIF1A expression inhibitor
		0.840	0.026	CYP2C12 substrate
		0.800	0.004	Chemopreventive
		0.805	0.010	TP53 expression enhancer
		0.753	0.018	Antineoplastic
3.	LIC	0.848	0.002	MMP9 expression inhibitor
		0.833	0.010	HIF1A expression inhibitor
		0.802	0.004	Chemopreventive
		0.773	0.014	TP53 expression enhancer
		0.723	0.004	AR expression inhibitor

**Table 4:** The chemical properties of the elucidated compounds.

Compound	Chemical Name	Molecular Formula	Molecular Structure
Glabrene	8-(7-hydroxy-2*H*-chromen-3-yl)-2,2-dimethylchromen-5-ol	C_20_H_18_O_4_	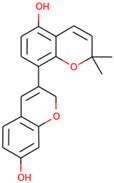
LIC	5,7-dihydroxy-3-[2-(2-hydroxypropan-2-yl)-2,3-dihydro-1-benzofuran-5-yl]chromen-4-one	C_20_H_18_O_6_	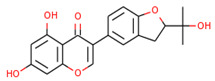

**Table 5:** The average values of different MD parameters calculated after 100 ns simulations.

System	RMSD (nm)	RMSF (nm)	Rg (nm)	SASA (nm^2^)	#H-Bonds
YES1	0.20	0.11	1.82	131	177
YES1-Glabrene	0.21	0.12	1.84	135	178
YES1-LIC	0.18	0.10	1.82	132	183

## Data Availability

All the data has been provided in the manuscript.
